# Influence of the Mediterranean Diet on Healthy Aging

**DOI:** 10.3390/ijms24054491

**Published:** 2023-02-24

**Authors:** Maria Carmen Andreo-López, Victoria Contreras-Bolívar, Manuel Muñoz-Torres, Beatriz García-Fontana, Cristina García-Fontana

**Affiliations:** 1 Endocrinology and Nutrition Unit, University Hospital Clínico San Cecilio, 18016 Granada, Spain; 2 Instituto de Investigación Biosanitaria de Granada (Ibs. Granada), 18014 Granada, Spain; 3 CIBER on Frailty and Healthy Aging (CIBERFES), Instituto de Salud Carlos III, 18012 Granada, Spain; 4 Department of Medicine, University of Granada, 18016 Granada, Spain; 5 Department of Cell Biology, University of Granada, 18016 Granada, Spain

**Keywords:** aging, Mediterranean diet, molecular pathways, microbiome

## Abstract

The life expectancy of the global population has increased. Aging is a natural physiological process that poses major challenges in an increasingly long-lived and frail population. Several molecular mechanisms are involved in aging. Likewise, the gut microbiota, which is influenced by environmental factors such as diet, plays a crucial role in the modulation of these mechanisms. The Mediterranean diet, as well as the components present in it, offer some proof of this. Achieving healthy aging should be focused on the promotion of healthy lifestyle habits that reduce the development of pathologies that are associated with aging, in order to increase the quality of life of the aging population. In this review we analyze the influence of the Mediterranean diet on the molecular pathways and the microbiota associated with more favorable aging patterns, as well as its possible role as an anti-aging treatment.

## 1. Introduction

Currently, the global population has a notably increased life expectancy compared to decades ago, exceeding 60 years of age in most cases. According to the World Health Organization (WHO), the percentage of people over 60 years of age will double globally by 2050 [1]. However, a longer life expectancy leads us to reconsider not only the health of older people but also what kind of implications aging has [2].

Aging is a natural physiological process that leads to a progressive loss of cellular functionality, with consequences that predispose people to an increased risk of frailty, morbidity, and mortality [3]. The role of lifestyle and diet can promote “healthy aging”, in which quality of life takes precedence. According to the WHO, this concept refers to the process of developing and maintaining a functional capacity that enables well-being in old age [1,4].

Several cellular and molecular hallmarks are involved in the aging process. In particular, there are nine hallmarks that are decisive in the aging process: genomic instability, telomere attrition, epigenetic alterations, loss of proteostasis, the dysregulation of nutrient sensing, mitochondrial dysfunction, cellular senescence, stem cell depletion, and altered intercellular communication [5]. These molecular mechanisms are involved in the development of age-related diseases such as cancer, obesity, diabetes, cardiovascular disease (CVD), and neurodegenerative diseases [3]. These age-related diseases have been associated with risk factors that can be modified mainly through nutrition, which constitutes one of the pillars of health [6]. In addition, the microbiota, which is modified by diet, has also been involved in aging [6]. It has been suggested that the age-related decline in immune system function (immunosenescence) and chronic low-grade inflammation could lead to microbiota disturbances that are associated with several age-related pathologies. Thus, it has been argued that a balanced diet can modulate the proliferation of specific bacteria within the gut microbiota. This has been associated with improved health status in older people [7].

In light of the above, the aim of our review was to analyze the available data regarding the potential effects of the Mediterranean diet (MedDiet) as a whole, or of individual elements of it, on the nine hallmarks of aging, to provide further evidence of its health benefits. Moreover, we have also reviewed the effect of MedDiet on the microbiota and its relationship to aging.

## 2. Aging and Frailty: Biological Links

From a biological point of view, aging can be defined as the physiological and progressive accumulation of senescent cells in organs and tissues, which occurs during the lifetime of an individual and leads to progressive functional slowing or the total loss of function [8,9,10].

Pleiotropic antagonist genes comprise a set of genes that regulate cellular senescence, performing an important role in preventing the degeneration of malignant cells in the cell cycle [11,12]. These genes are also involved in protective mechanisms in physiological cellular senescence processes and in age-related diseases. However, aging cells produce proinflammatory and lytic extracellular matrix molecules in a process known as the senescence-associated complex secretory phenotype (SASP), resulting in degeneration and pathological senescence. Moreover, the aging process involves the immune system; in particular, the cell-mediated defense mechanism is slowed down. Senescent cells do not produce sufficient signals to activate immune cells. Likewise, senescence is induced by the accumulation of various factors at the cellular level that is responsible for macromolecular damage, such as secondary DNA alterations due to oxidative damage, telomere shortening, and endoplasmic reticulum (ER) degeneration [13]. Thus, aging is the result of multifactorial interactions between local and systemic environmental factors and involutional factors due to cellular senescence. Therefore, the number of senescent cells in a person’s body increases with age as the aging immune system becomes less efficient and senescent cells accumulate. This makes individuals more vulnerable to further deterioration after exposure to environmental stressors [13]. The disease occurs when environmental stressors attack tissues that are already in the presence of senescent cells with very low resilience [14,15].

Frailty develops due to an increasing decline usually linked to age, severe deterioration, and the onset of pathological states. This leads to a condition of increased vulnerability and reduced adaptive capacity, and ultimately, negative health changes are triggered by even mild stressors. It is considered more appropriate to speak of “frailty syndrome”: a chronic pathological condition resulting from the interaction between several factors, including aging-related physiological alterations, pluripathology, nutritional deficiencies up to severe malnutrition, and the negative impact of socio-environmental factors [16]. In fact, a high proportion of undernourished people are frail, and undernutrition leads to weight loss, which can contribute to frailty syndrome [17]. At the other extreme, obesity increases the risk of frailty [18]. In terms of body composition, frailty has been associated with a higher body fat mass and fat percentage and with a low muscle mass and is often without association with the body mass index [19,20,21].

All of this can lead to the frail elderly losing all self-sufficiency, increasing the risk of falls, and can result in a state of confusion with severe impairment of cognitive functions that ultimately increases the risk of the development of diseases [22].

## 3. The Mediterranean Diet

The term MedDiet was first coined by Ancel Keys in the 1960s [23]. The MedDiet reflects the dietary patterns typical of civilizations based around the Mediterranean Sea, especially Greece, the island of Crete, and southern Italy in the early 1960s [24]. In fact, the MedDiet is closely linked to traditional olive growing areas in the Mediterranean region and has been associated with low rates of chronic diseases (lower risk of CVD and metabolic diseases associated with excess weight) and, consequently, high life expectancy [24,25].

The MedDiet is characterized by a high consumption of olive oil (OO) as the main source of fat—especially virgin (VOO) and extra virgin (EVOO)—and the high consumption of plant foods (vegetables, fruits, legumes, potatoes, bread, and other cereals (minimally refined), nuts and seeds), as well as fresh seasonal, locally grown, and minimally processed foods. Dairy intake is moderate (mainly cheeses and yogurts), and fish (an excellent source of long-chain poly-unsaturated fatty acids (PUFAs), particularly omega-3) and poultry are consumed in low or moderate amounts. The MetDiet includes the low consumption of red meat and sweets and moderate consumption of wine at meals. No more than four eggs are consumed per week. In general terms, caloric intake in the form of fat does not exceed 30% of the intake, with less than 8–10% contributed by saturated fats. Some bioactive compounds in the MedDiet include vitamins, minerals, polyphenols, fiber, nitrates, PUFAs, and mono-unsaturated fatty acids (MUFAs) that, in combination or separately, are beneficial to health [26,27,28]. Among the PUFAs, the essential omega-6 fatty acid is linoleic acid (LA). In addition, longer omega-3 PUFAs, such as eicosapentaenoic acid (EPA) and docosahexaenoic acid (DHA), are derivatives of alpha-linoleic acid. These are mainly present in fish oils [29]. For these reasons, MedDiet is unique and different from other healthy dietary patterns [29].

## 4. Interplay between Mediterranean Diet, Aging and Frailty

### 4.1. Mediterranean Diet and Hallmarks of Ageing

**Figure 1:**
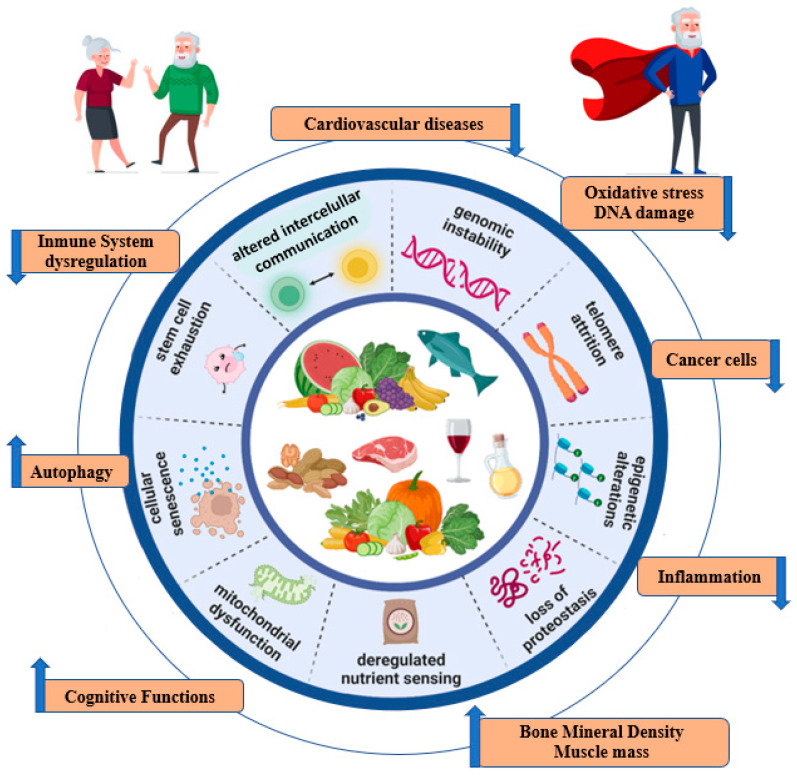
Impact of MedDiet on the hallmarks of aging.

The aging process has been linked to nine distinctive cellular and molecular features [5] (Figure 1). Each of these plays its role in the trajectory of natural aging; their experimental exacerbation accelerates the process, and their optimization slows it down, thus increasing lifespan [30]. External lifestyle factors such as diet can modulate the aging process [31,32].

#### 4.1.1. Genomic Instability

Aging increases susceptibility to DNA alterations resulting from a combination of oxidative stress, epigenetic alterations, damaged DNA, and telomere attrition [33]. Unrepaired DNA could increase the risk of mutations and favor the beginning or development of age-related diseases [34,35]. The MedDiet could play a protective role against genomic alterations. Indeed, bioactive compounds that are contained in the MedDiet, such as melatonin, phytosterols, carotenoids, polyphenols (such as resveratrol and hydroxytyrosol (HT)), vitamins, and glucosinolates (in cruciferous vegetables), can promote DNA repair and attenuate telomere shortening [36,37]. These positive effects have been explained by the anti-inflammatory effects of the MedDiet and the direct and indirect (epigenetic) modifications induced by the MedDiet on gene expression [38,39,40,41].

DNA damage due to oxidative stress is a result of the failure of oxidative damage-repair mechanisms as a result of excess reactive oxygen species (ROS) [42]. Guanine is an important target for DNA oxidation, generating oxidized metabolites. Among them, 8-oxo-2′-deoxyguanosine (8-OHdG) is considered a marker of oxidative stress with mutagenic potential [43,44,45]. Urquiaga et al. related the MedDiet, including a moderate intake of red wine, with a reduction in 8-OHdG levels in the peripheral blood leukocyte DNA, which impacts positively on the control of oxidative stress [46]. Similarly, lower levels of another marker of oxidative stress, the deoxyguanosine adduct, were associated with greater adherence to the MedDiet in the Italian cohort of the EPIC (European Investigation into Cancer and Nutrition) study [47].

Nuts and cooked tomato sauce using OO highlight the protective role against oxidative DNA damage [48,49,50]. This defense phenomenon was found in intervention studies in the human population after the consumption of tomato [51,52], broccoli [53,54], spinach [55,56], and blueberries [57,58]. In addition, a study in rats fed with a diet enriched with VOO found an association with less damage in the genetic material in peripheral blood cells vs. rats fed with sunflower oil [59]. In this context, MUFAs and PUFAs have been demonstrated to play a protective role against oxidative stress and DNA damage [60,61,62]. In a sub-analysis of the PREDIMED study, the intervention group with the MedDiet with MUFA (EVOO) or PUFA (walnuts) compared to the control (low-fat diet) showed a significant improvement in cardiovascular outcomes and a lower proportion of oxidative markers in urine [63]. Likewise, another clinical trial demonstrated better control of atherosclerosis markers in individuals on the MedDiet consuming OO; however, only the group whose main fat source was VOO decreased urinary 8-OHdG levels [64]. In general, the MedDiet and OO intervention groups showed positive results in the regulation of DNA repair genes. In fact, a low expression of the polymerase k gene, which encodes a protein that is responsible for replicating damaged DNA, was identified in the MedDiet and OO intervention groups [64]. Thus, this dietary pattern showed a favorable effect on blood pressure, insulin sensitivity, and lipid levels exerted in people with a high cardiovascular risk [65,66,67,68,69].

Modifications in dietary habits towards the Mediterranean pattern have been proposed as positive in the reduction in oxidative DNA damage in cancer patients [70]. In addition, several studies indicate an inverse association between adherence to the MedDiet and very prevalent neoplasms such as breast cancer, colorectal cancer (CRC), bladder cancer, or prostate cancer [71,72,73]. Specifically, individuals with CRC and high adherence to the MedDiet presented a lower histological grade and a lower frequency of synchronous adenomas compared to oncologic patients with low adherence to the MedDiet. In fact, elevated values of glutathione peroxidase (with antioxidant properties) and decreased 8-OHdG values were detected in patients with higher adherence to the MedDiet [74].

Regarding neurodegenerative diseases, a decreased risk of Alzheimer’s disease (AD) was associated with the direct effect of MedDiet components on the pathogenesis of AD [75,76]. The beneficial effects of this neurodegenerative disorder were primarily due to EVOO consumption. Oleuropein (one of the main phenolic components of green olive pulp) reduced Poly ADP-ribose polymerase (PARP)-1 activation, protecting neuronal cells from oxidative damage. Furthermore, Zhang et al. observed an age-dependent increase in 8-OHdG in human brain tissues, particularly in those belonging to people with AD [77]. In people with mild cognitive impairment, an increase in 8-OHdG was detected in certain brain areas, and this could be interpreted as a biomarker predictive of AD pathogenesis [78]. Thus, when evaluating oxidative DNA damage according to 8-OHdG values in patients with mild cognitive impairment who consumed EVOO for one year, 8-OHdG levels were reduced [79].

Overall, it seems that the Mediterranean pattern has potentially positive effects on genomic instability.

#### 4.1.2. Telomere Attrition

Telomeres are nucleotide sequences at the ends of chromosomes, which progressively shorten with age. They are known as biomarkers of aging. Chronic oxidative stress is associated with telomere attrition [80]. In addition, inflammation stimulates telomere shortening by increasing the rate of hematopoietic stem cell replication to supply the leukocyte demand generated in the inflammatory process [81]. Although telomere length is inherited, it is influenced by external factors such as smoking, obesity, and a sedentary lifestyle. In turn, telomere shortening increases the risk of CVD, cancer, and mortality, especially at early ages [82,83].

Greater adherence to the MedDiet was associated with longer telomeres and greater telomerase activity [84,85], which could relate to lower levels of inflammation and oxidative stress [84,86]. In their meta-analysis, Canudas et al. established a positive association between MedDiet adherence and telomere length in the blood cells except for samples taken from men [87]. However, it should be noted that the included studies were cross-sectional and, therefore, did not establish causality. Nevertheless, this result can be contrasted with the two prospective studies conducted to date [88,89]. The work of Meinilä et al. prospectively studied 1046 Dutch subjects with a mean age of 61 years over a period of 10 years and did not establish an association between the male sex and adherence to the MedDiet. In the same study, women had faster telomere shortening [89]. On this subject, the only clinical trial found to date was by García-Calzón et al.; this was a sub-analysis of the PREDIMED-Navarra study, which evaluated 520 individuals, comprising 55% women aged 55–80 years. Three intervention groups were randomly assigned to a control or low-fat diet and there were two MedDiet groups, one supplemented with EVOO and the other with mixed nuts. In the cross-sectional analysis at the baseline, better adherence to the MedDiet was associated with longer telomeres only in women. However, assignment to the MedDiet-nuts group was associated with a higher risk of telomere shortening 5 years after the intervention, with no differences for the group supplemented with EVOO [88]. There was no consistent explanation for these findings, although cohort variables such as ethnicity, genetics, or sex could be related. In the work of Gu et al., a positive association was found between MedDiet adherence and telomere length in non-Hispanic white people [90]. This association was not found in African Americans or Hispanics [90]. However, in another sub-analysis of the PREDIMED-Navarra study, it was shown that the Pro12Ala polymorphism in the peroxisome proliferator-activated receptor γ2 (PPARγ2) gene interacted with MedDiet to prevent telomere shortening [91].

Regarding PUFAs, omega-3 was better than omega-6 since attenuated telomere shortening was shown in a cohort of people over 65 years of age with mild cognitive impairment supplemented with this kind of fatty acid [92]. Similarly, the Breitas–Simoes clinical trial demonstrated attenuation in telomere attrition in cognitively unimpaired elderly people who supplemented their usual diet with walnuts (a source of omega-3 PUFAs) for 2 years [36]. However, LA supplementation in a population of 299 elderly people with recent myocardial infarction was associated with increased leukocyte telomere length, and no relationship was established with other PUFAs [93]. In general, it can be concluded that it is beneficial to follow a healthy diet in which PUFAs are present.

Telomere length has been linked to several types of cancer. The finding of short telomeres in CRC suggests that telomere shortening contributes to tumorigenesis and the genetic instability of premalignant cells. In fact, severely short telomeres have been shown to cause senescence in healthy cells or genomic instability in premalignant cells [83,94,95]. Indeed, altered telomere length homeostasis and unrepaired DNA damage were considered key in the onset, progression, and prognosis of CRC [95]. In this regard, the MedDiet could be useful primarily as a preventive therapy since several studies show the association between good adherence to the MedDiet and telomere preservation [84,91].

In addition, telomere shortening has been related to neurodegenerative diseases [96,97]. In fact, Guo et al. suggested that telomere length has a causal effect on the risk of AD due to oxidative stress and inflammation [97]. Furthermore, telomere shortening has been associated with cognitive impairment, amyloid pathology, and tau protein hyperphosphorylation in AD [96]. In this regard, the oleuropein aglicone from OO inhibits protein aggregation in AD [98]. Further, the Mediterranean diet could prevent telomere shortening after oxidative damage thanks to antioxidant-rich vegetables such as nuts and seeds [99]. Resveratrol (an antioxidant present in grapes) generated neuroprotective effects in a study on a mouse model of AD [100].

In summary, increased adherence to the MedDiet could attenuate telomere attrition. However, these beneficial effects could be limited to specific subgroups of the population. Further studies are needed to resolve the controversies raised.

#### 4.1.3. Epigenetic Effects

Epigenetics encompass inherited genomic changes that occur in the absence of direct DNA damage. Young, healthy individuals maintain compact chromatin and the optimal epigenetic regulation of biological processes. However, aging favors the accumulation of chromatin damage, which compromises genome integrity and alters cellular function [101]. DNA methylation (mDNA) is considered one of the best-known epigenetic markers. mDNA is used as a clock for the calculation of biological age [102]. The long interspersed nuclear element (LINE-1) was used as a marker of global mDNA because it is the most common repetitive sequence in the human genome, and 1/3 of mDNA occurs in LINE-1 [103,104]. Specifically, the hypomethylation of LINE-1 occurs during aging and is associated with multiple cancers and CVD [105]. In addition, oxidative stress plays a role in mDNA via the carbon cycle [42], so the more ROS there is, the greater the DNA damage; finally, DNA undergoes hypomethylation to defend itself [106]. In this context, a diet rich in antioxidants, such as the Mediterranean diet, is proposed as an epigenetic diet.

To date, several studies have linked MedDiet adherence to LINE-1 hypomethylation. Specifically, there are two clinical trials that establish an inverse relationship between these two factors [107,108]. In the work of Agodi et al., women of childbearing age with low adherence to the MedDiet, particularly those with lower fruit intake, were at higher risk of LINE-1 hypomethylation [109]. This finding may be related to the fact that fruit is a folate-rich food, and folate is an important donor of methyl groups. Other more specific components of the MedDiet, such as nuts and EVOO, are able to induce methylation changes in several peripheral white blood cell genes related to diabetes, inflammation, and signal transduction, which may have potential health benefits [110]. Similarly, the MedDiet could contribute to delaying the process of carcinogenesis that is related to DNA methylation processes since several studies have shown lower LINE-1 methylation levels in different types of tumors, such as CRC or breast cancer [111,112].

Regarding CVD, the review published by Muka et al. supported the suggestion that global mDNA, according to repeated LINE-1 hypomethylation, is inversely associated with CVD risk independently of established cardiovascular risk factors [105]. In general terms, LINE-1 hypomethylation is linked to an unfavorable cardiovascular risk profile due to its association with diabetes, obesity, lower HDL-cholesterol levels, elevated total cholesterol levels, and inflammation [113,114].

However, the expression of RNA (ribonucleic acid) contributes to the epigenetic modulation of gene expression that alters cellular functionality. In this context, the overexpression of miR-155-3p has been linked to carcinogenesis [115]. In fact, Ping Li et al. detected an increased expression of miR-155-3p in relation to CRC tumor growth [116]. In addition, Let-7b, a regulator of histone H2B ubiquitination, showed a probable antitumor effect [117]. In respect of this line, Li et al. showed that let-7b-3p inhibited tumor growth and metastasis in lung cancer, correlating with the low expression of this molecule with poor prognosis in lung adenocarcinoma patients [118]. As a preventive therapeutic strategy, good adherence to the MedDiet could decrease the expression of miR-155-3p and increase the expression of let-7b-3p, improving the risk and evolution of cancer [119].

In relation to neurodegenerative diseases associated with aging, epigenetic modifications play important roles in AD [120]. Specifically, mDNA is a highly controlled mechanism involving nicotinamide adenine dinucleotide (NAD)-dependent deacetylase Sirtuin 1 (SIRT1) that prevents altered methylation [121]. In this regard, Luccarini et al. demonstrated that quercetin and other EVOO polyphenols activate the SIRT1 pathway with suggestive therapeutic and preventive benefits [122,123].

Therefore, it is likely that there is an age-related disease—epigenetic interaction that may benefit from a healthy Mediterranean pattern diet.

#### 4.1.4. Proteostasis

Proteostasis, or protein homeostasis, refers to the work of a complex network of pathways that are essential for cell function and viability, ensuring the appropriate concentration, folding, and interactions of proteins from synthesis to degradation [5]. Specifically, chaperones and two proteolytic systems (the ubiquitin-proteasome and the lysosome-autophagy system) are responsible for the maintenance of proteostasis [124]. The progressive loss of cellular protein homeotasis is detected during aging, and proteomes that are more stable or more resistant to alterations are found in the longest-lived species [125].

The age-related deterioration of proteostasis affects chaperone functionality due to the cellular energy deficit that is inherent to senescence [126]. In addition, autophagy and the proteasome are altered with age, influencing proteotasis [127,128]. In this regard, experimental interventions that enhanced autophagy-activating properties were associated with healthier aging [129]. Dietary habits could be beneficial for the optimization of proteostasis. Indeed, MedDiet polyphenols such as resveratrol can directly activate autophagy [129]. Likewise, oleuropein has been highlighted as an autophagy enhancer through a protein mammalian target of rapamycin (mTOR) and the adenosine monophosphate-activated protein kinase (AMPK)-dependent mechanism [130]. Furthermore, the antioxidant properties of these MedDiet components could attenuate the excess of oxidized proteins associated with senescence and age-related diseases [131].

Age-related diseases such as neurodegenerative diseases (in particular, AD and Parkinson´s disease, PD) have been related to the deterioration of proteostasis [132]. In AD, hyperphosphorylated tau protein was aggregated abnormally and created insoluble neurofibrillary tangles, which were involved in neurodegeneration [133,134]. In addition, amyloid-beta (Aβ) peptide accumulated and formed plaques that damaged neuronal cells [135]. In relation to PD, aggregates of insoluble α-synuclein protein fibrils were present in the neurons of people with PD and were neurotoxic [136]. Overall, this loss of proteostasis in neurodegenerative diseases is closely linked to inflammation and cellular senescence [13,137]. Shannon et al. proposed the MedDiet as a mechanism by which to prevent neurodegeneration due to its modulating effect on protein homeostasis [30]. By enhancing autophagy, OO could mitigate the effects of toxic vascular agents, favoring the prevention of late-onset AD [29]. Specifically, oleocanthal could reduce tau protein polymerization [138]. The interaction between oleocanthal and tau proteins induces a tau rearrangement that may explain the antifibrillogenic effect of oleocanthal [139]. Thus, oleocanthal intervention in mice has been shown to increase the yield in the activity of blood–brain barrier transporter proteins that remove Aβ peptides (P-glycoprotein and low-density lipoprotein receptor-related protein 1). Thus, the percentage of degraded Aβ peptides was higher in the treated group [140]. The beneficial effect of oleocanthal in mice was extensible to human cell lines since an improvement in Aβ transport by Aβ-secreting cells was observed after administering oleocanthal for 72 h [141]. Further, resveratrol reduced β-secretase activity and Aβ-peptide aggregation in AD murine models and acted as a neuroprotectant in AD and PD [142].

In relation to CVD, alterations in protein homeostasis and stability in the proteome can influence healthy cardiac aging [143]. The increased accumulation of misfolded protein aggregates was detected in CVD by the downregulation of the HSP70 chaperone in vascular tissue during aging [144,145]. In general terms, decreased proteasome activity was detected in the atherogenic plaques of aged rats and elderly patients [124,146]. In this context, oleuropein raised the rates of proteosome-mediated degradation in human fibroblast cultures, their lifespan was increased, and senescence was delayed by 15% [147]. A diet rich in EVOO also increased messenger RNA levels of the autophagy marker LC3 in older rats compared to rats that were fed using other sources of dietary fat [148].

Urra et al. identified altered proteostasis as a hallmark of cancer. The hypermetabolism of cancer cells and the overexpression of oncogenes were related to ER stress. In response to this stress, the unfolded protein response (UPR) was generated [149]. Therefore, the UPR functioned as an adaptive mechanism during cancer progression [149]. Nevertheless, the activation of UPR at different stages of cancer evolution experienced a complex progression [149]. The role of the UPR during the early phase of cancer development prevented oncogene-induced malignant progression [150]. Cells surviving oncogene-induced apoptosis elevated UPR activation levels. Thus, in later stages of cancer progression, the UPR modified part of its function and contributed to tumor growth, aggressiveness, microenvironment adaptation, and resistance to treatment [149]. In fact, in human biopsies of breast cancer, lymphoma, or multiple myeloma, x-box-binding protein-1 (XBP-1), a protein that signals the activation of the UPR complex, was highly expressed and correlated with poor prognosis [151,152,153,154]. In addition, the IRE1α-XBP1 UPR signaling pathway was linked to the promotion of triple human breast [155], prostate [156], and hepatocellular cancers [157]. In this sense, MedDiet could prevent the perpetuation of this process. The work of Yubero-Serrano et al. observed that the MedDiet decreased the expression of genes relating to endoplasmic erythrocyte stress, such as XBP1 [158]. In addition, OO MUFAs “colonized” cell lipid membranes, which was associated with reduced susceptibility to ER stress and apoptosis [159]. In addition, secoiridoids such as oleuropein from EVOO favored the turnover of misfolded proteins in the cell by promoting compensatory UPR activity. Nevertheless, these bioactive compounds were able to inhibit the growth of highly aggressive mammary malignant cells [160,161].

Overall, the prevention of neurodegenerative, CVD, and oncological diseases may be of great interest given the proteostasis-activating role played by some components of the MedDiet. Nevertheless, further studies are needed to clarify the role of specific MedDiet nutrients in the prevention of these pathologies.

#### 4.1.5. Nutrient-Sensing Pathways

These pathways are signaling systems responsible for detecting the availability of cellular resources that are essential for maintaining functionality, growth, and reproduction and thus participate in the aging process [162]. In fact, it is hypothesized that the proper regulation of these signaling pathways may extend lifespan and decrease the risk of age-related diseases [5].

In particular, the dysregulation of some nutrient-sensing pathways such as insulin/insulin-like growth factor-1 (IIS), mTOR, AMPK, and sirtuins was linked to an increased risk of non-inheritable diseases associated with age [5,163]. The IIS pathway is the most conserved aging control pathway in evolution, and it regulates glucose metabolism [5]. Multiple genetic polymorphisms or mutations that reduce the intensity of signaling in the IIS pathway were associated with an increased lifespan in mice [164]. Paradoxically, however, in physiological or pathological aging, growth hormone (GH) and insulin-like growth factor-1 (IGF-1) levels decrease [165]. This phenomenon is explained as a defensive response of the organism to minimize cell growth and metabolic response in a scenario of systemic damage [166]. Nevertheless, low concentrations of IGF-1 at the peripheral level are associated with an increased risk of type 2 diabetes mellitus, CVD, sarcopenia, osteoporosis, and frailty in elderly humans [167,168]. This has been explained by the decrease in insulin sensitivity that appears with age [168]. However, reduced IIS pathway signaling stimulates longevity-related Forkhead Box O (FOXO) proteins, which improve mitochondrial function and promote glucose metabolism through lipid oxidation [169]. In this regard, Calnan et al. proposed to control FOXO activity by up-regulating the expression of genes involved in resistance to metabolic stress and apoptosis to promote healthy aging [170].

Together with the IIS pathway, mTOR is the main accelerator of aging [5]. The mTOR pathway identifies high concentrations of amino acids, and its regulation has been associated with healthy aging. Specifically, mTOR is a kinase that is formed by two protein complexes: mTORC1 and mTORC2 [171]. In mice with low levels of mTORC1 or S6K1 (the ribosomal S6 protein kinase 1) activity (main substrate of mTORC1) and normal levels of mTORC2, increased life expectancy was detected [172,173]. The hyperstimulation of this pathway was frequently observed in diseases associated with aging, such as cancer [174,175], AD [176], and diabetes [177]. However, the inhibition of mTOR activity also showed undesirable effects, including wound healing problems, insulin resistance, and testicular degeneration in mice [178].

**Figure 2:**
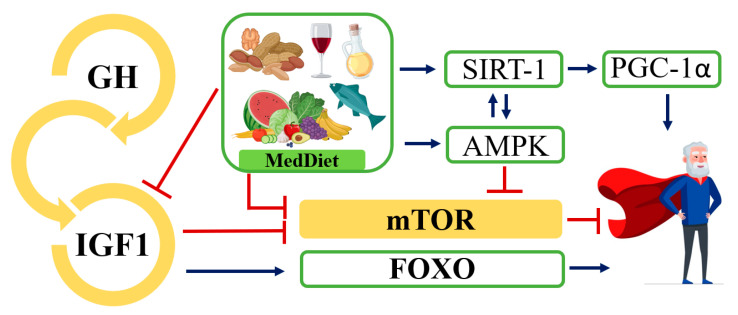
Dysregulated nutrient sensing pathways. Overview of the somatotropic axis, involving GH and the IIS pathway. Molecules that promote aging are shown in yellow, while molecules with anti-aging properties are shown in light green. Red lines indicate inhibited or slowed pathways and blue lines indicate activated pathways that together promote healthy aging. GH, growth hormone; IGF-1, insulin-like growth factor-1; mTOR, protein mammalian target of rapamycin; AMPK, adenosine monophosphate-activated protein kinase; FOXO, Forkhead Box; PGC-1α, peroxisome proliferator-activated receptor gamma 1-alpha; SIRT-1, sirtuine-1.

In contrast to the IIS and mTOR pathways that sense nutrient abundance and favor anabolism, AMPKs, and sirtuins sense nutrient scarcity and promote energy catabolism. In fact, the activation of AMPK promoted longevity by the inhibition of one mTOR complex, mTORC1 [179], and one of the sirtuins, SIRT1, could have triggered peroxisome proliferator-activated receptor gamma 1-alpha (PGC-1α) coactivator upon deacetylation [180,181]. This coactivator is involved in the transcription of antioxidant genes and is a key regulator of mitochondrial biogenesis [182] (Figure 2).

In animal models, a dietary restriction has been shown to promote healthy aging mediated by nutrient-sensing pathways [183]. However, in humans, less stringent and more realistic interventions are being proposed [163]. In this regard, MedDiet, characterized by low-moderate protein intake, low glycaemic index, and polyphenol-rich foods, may be an alternative [184]. Polyphenol-rich foods activate the AMPK and sirtuin pathways, while mTOR is inhibited and autophagy is stimulated [185,186]. Interestingly, oleocanthal exhibited potent neuroprotective and antitumoral properties by inhibiting mTOR activity [187]. Antiproliferative effects were observed in certain breast cancer cell lines, although this effect was not fully clear in other neoplasms, such as CRC and cervical cancer, probably due to the lower expression of the mTOR pathway in these two malignant processes [141]. Moreover, oleuropein and HT showed an antidiabetic effect in addition to attenuating oxidative stress in rats with diabetes mellitus [188,189]. Further, adherence to the MedDiet may be of interest to patients with AD. In these cases, neurons presented a reduced activity of glucotransporters, GLUT1 and GLUT3, leading to altered insulin signaling [190], altering fasting blood glucose along with lipid metabolism dysfunction [191]. In addition, low IGF-1 values were detected with a low-glycemic-index diet compared with a high-glycemic-index diet [192], which could attenuate the signaling intensity of the IIS pathway favoring longevity.

However, in the study of Levine et al., people aged from 50 to 65 years who ingested more protein (above 20% of the daily caloric intake) presented higher mortality, and 25% of deaths were associated with cancer [193]. Those individuals with moderate intake recorded a lower IGF-1 concentration by down-modulating the activity of the IIS and mTOR pathways [192,194]. In addition, high protein intake was linked to an increased risk of type 2 diabetes, obesity, and CVD [195]. Nevertheless, the protein quality is important. In this context, in murine models, methionine restriction showed a prolonged life expectancy and protection against multiple chronic diseases, particularly cancer [196]. Other essential branched-chain amino acids (in poultry, dairy, and eggs) such as leucine, isoleucine, and valine were identified as key in the regulation of insulin sensitivity via mTOR [197]. In fact, Fontana et al. reported that selective reduction in the dietary intake of branched-chain amino acids improved glucose tolerance, β-cell metabolic stress, and body composition [198].

Accordingly, there is robust evidence that some components of the MedDiet have properties that favor healthy aging, especially EVOO, so it would be interesting to further explore this through its action on nutrient signaling pathways.

#### 4.1.6. Mitochondrial Dysfunction

Mitochondria are cellular organelles that are responsible for producing much of the adenosine triphosphate (ATP) necessary for cell survival and modulating signaling toward apoptosis. Much of the total oxygen taken up by cells is metabolized in the electron transport chain located in the inner membrane of the mitochondria. The formation of ROS at this level represents potential intracellular damage to the organelle. In general, mitochondria are altered by oxidative damage with aging, and their deterioration is a consequence of the tissue’s inability to repair or eradicate the damage [199]. In the elderly, an excess of dysfunctional mitochondria can result in less energy in the form of ATP and more ROS than in the younger populations [5]. Indeed, this mitochondrial imbalance has been associated with age-related neurological diseases such as PD and AD [200,201], cancer [202], and metabolic syndrome [203].

Mitochondrial dysfunction is also implicated in the pathophysiology of type 2 diabetes mellitus, obesity, dyslipidemia, and CVD [203]. These pathologies are usually described as metabolic syndrome. Impaired mitochondrial energy metabolism is considered the main cause of metabolic syndrome [203]. Specifically, in type 2 diabetes mellitus, high glucose levels increase ROS production with consequent damage to the mitochondria [204] and inhibition of the IIF pathway, which favors lipid accumulation leading to metabolic disorders [205,206,207,208,209,210]. Aging, the alteration of mitochondrial antioxidant effects, and genetic factors promoting insulin resistance are the main causes of many metabolic diseases [203]. In this sense, the MedDiet with MUFAs and antioxidants may have a beneficial effect. In fact, different experimental models have demonstrated that components of MedDiet, such as polyphenols, plant-derived compounds, and PUFAs, could correct mitochondrial dysfunction and improve mitochondrial metabolism [211]. Indeed, the PREDIMED study demonstrated the cardiometabolic benefits [212].

Varela-López et al. demonstrated that OO has a favorable effect on the mitochondrial structure and function of aged rats [213]. Along the same lines, MUFA-rich OO played a key role in adapting the lipid profile of mitochondrial membranes to provide resistance against oxidative damage and dysfunction associated with aging [141]. In fact, the predominant dietary fat source impacted mitochondrial membrane biochemistry by modifying the fatty acid composition profile and electron transport systems [214]. In addition, PUFA sources favored the oxidation of mitochondrial membranes compared to saturated or MUFA sources [215]. In this sense, Ochoa et al. measured the mitochondrial fatty acid profile, catalase activity, and hydroperoxide levels in the liver, heart, and skeletal muscle of Wistar rats when supplemented with different fat sources (sunflower oil versus OO) [216]. Those with higher OO intake had more MUFAs in their mitochondrial membranes, and those fed mainly with sunflower oil had more omega-6 PUFAs. These variations were consistent with the levels of oxidative damage, i.e., those fed OO had fewer hydroperoxides in their body tissues compared to those who were fed sunflower oil. Therefore, a diet rich in OO generated membranes with fewer PUFAs, which attenuated an increase in age-related lipid peroxidation in post-mitotic tissues such as the heart and skeletal muscle [217]. Furthermore, in the liver (the prototype of regenerative tissue) and heart, a greater increase in catalase activity—essential for antioxidant defense in relation to life expectancy—was observed in rats who were fed a diet rich in OO [218,219].

However, HT and oleuropein reduce oxidative stress and optimize mitochondrial function [220,221]. HT improves neuronal inflammation and may delay the development of AD [222]. In fact, mitochondrial dysfunction is critical in the early stages of AD and PD, and the antioxidant power of wine polyphenols can protect organelles [200,223,224]. Quercetin and procyanidins (the main polyphenols in wine) can decrease ROS and improve the cell viability of neuronal and astrocytic cell lines [225,226]. Specifically, quercetin decreases ROS production through the overexpression of the AMPK/SIRT1 signaling pathway [227]. Resveratrol improved the antioxidant status in PD rats and reduced dopamine loss [228]. However, the mechanism by which resveratrol protects mitochondrial function and homeostasis is not fully understood but has been investigated for its potential applications in the treatment of age-related diseases [229]. Overall, several polyphenols are present in wine and individually carry out promising mitochondrial protection. However, Kurin et al. demonstrated greater antioxidant potency when combining several polyphenols with respect to their individual activity [230]. Thus, light to moderate wine intake in humans can favor the expression of antioxidant enzymes in the blood [231]. Although most of the studies evaluating the impact of MedDiet components on mitochondrial dysfunction were conducted in animals, they are also consistent with those conducted in humans.

Fish oil, a high-omega-3 PUFA source, has a protective effect on age-related mitochondrial dysfunctions similar to that observed for OO. Afshordel et al. demonstrated that fish oil supplementation for 21 days restored the concentration of omega-3 PUFA derivatives, improving mitochondrial function and consequent ATP synthesis in the brains of older mice [232]. The benefits of omega-3 PUFAs in neurodegenerative diseases have been observed in preclinical studies, while most of the controlled clinical trials have not met expectations. In this regard, initiating clinical work prematurely in the course of the disease and increasing the durability of the study may be helpful in obtaining the expected outcomes [233]. In addition, in cerebral ischemia, DHA showed beneficial results, and the reduction in stroke events was related to less disruption of the blood–brain barrier, less brain edema, and less inflammatory cell swelling [234].

Cell membranes with high concentrations of peroxidized PUFAs, which are typical of aging, led to apoptosis and growth inhibition [202]. In fact, the main products of lipid peroxidation are toxic and mutagenic aldehydes such as malondialdehyde (MDA) and 4-hydroxynonenal/4-hydroxy-2-nonenal (HNE). In addition, elevated MDA values were recorded in the plasma and blood of patients with breast, lung, and ovarian cancer [235,236,237,238,239]. In the work of Li YP et al., HNE promoted breast cancer cell growth and angiogenesis [240]. In this sense, the antioxidant capacity of OO could attenuate peroxidation, preventing or defending against the activation of carcinogenesis [217]. In fact, EVOO could have a beneficial effect on breast cancer risk [241].

In conclusion, mitochondrial dysfunction and oxidative damage play a crucial role in the pathogenesis of aging and longevity-related diseases. Together, different components of the MedDiet, such as OO, PUFA, and red wine, contribute to the maintenance of mitochondrial function. However, more studies are needed to evaluate the synergistic effect of MedDiet components on this hallmark.

#### 4.1.7. Cellular Senescence

Senescent cells often exhibit irreversible DNA damage, leading to cell cycle arrest. In addition, these cells produce a proinflammatory secretome or SASP that contributes to aging [242,243]. Cellular senescence is associated with other features of aging, including mitochondrial dysfunction, autophagy disorders, altered nutrient signaling, and epigenetic effects [5,244]. Overall, age increases the number of senescent cells, which increases the likelihood of age-related diseases [245]. This hallmark defends tissues from damaged and potentially oncogenic cells [5]. However, it requires progenitor cells with a regenerative capacity to compensate for the cellular deficit associated with aging [5].

Similar to DNA damage, exaggerated mitogenic (senescence-inducing) signaling is the other stress that is strongly associated with senescence. There are important cellular mechanisms that defend an organism against oncogenic or mitogenic alterations, such as the p16 INK4a/Rb and p19 ARF/p53 pathways [246]. Indeed, p16 INK4a (and, to a lesser extent, p19 ARF) levels correlate with age in most of the tissues analyzed [247,248]. In a meta-analysis performed by Jeck et al., the INK4a/ARF genomic locus was found to be the locus most closely linked to age-associated pathologies, including several types of CVD, diabetes, glaucoma, and AD [249].

Senolytic therapies, including dietary intervention, may delay or prevent cellular aging [250,251]. In fact, the MedDiet has demonstrated senolytic properties thanks to various food components. For example, nuts and certain vegetables seem to prevent the accumulation of senescent cells [53,55,252]. Further, the phenolic components of EVOO (oleocanthal or oleuropein), with antioxidant and anti-inflammatory effects, could play a relevant role in neurodegenerative diseases such as AD [253,254,255]. Specifically, tauopathy was associated with astrocyte or microglia senescence [256,257] and oleocanthal-reduced tau protein polymerization [138]. In addition, the Aβ peptide was identified as a potent inducer of cellular senescence [258,259,260,261,262]. The intervention with oleocanthal in mice showed increased performance in the activity of blood–brain barrier transporter proteins in charge of eliminating Aβ peptides (P-glycoprotein and low-density lipoprotein receptor-related protein 1), with the percentage of degraded Aβ peptides being higher in the treated group. The beneficial effect of olocanthal in mice may be extensible to human cell lines [141].

Resveratrol can delay or prevent senescence, as proven in human cell models (mesenchymal stem cells) [263,264]. Further, quercetin, when associated with Dasatinib (BCL family apoptotic inhibitors) in people with diabetic nephropathy, reduced the number of senescent cells in human adipose tissue [265]. However, there is clinical evidence of an association between age-related cardiac pathologies and the release of SASP components by senescent cells. The heart diseases that have been studied are heart failure, ischemia and myocardial infarction, and cardiotoxicity secondary to cancer chemotherapy [266]. However, the specific role of senescent cells in these conditions is unclear, and existing information is contradictory [266]. Presumably, the presence of maintained (and not transient) cellular senescence promotes deleterious effects in cardiac disease, such as the functional impairment of cardiac progenitor cells [267]. In addition, this hallmark can impair adult cardiomyocyte proliferation [267]. In this case, the MedDiet could reduce the production of proinflammatory substances since resveratrol inhibits the nuclear transcription of factor κB (NF-κB), which is essential for the genesis of SASP [268,269]. In addition, quercetin (associated with dasatinib) can facilitate programmed senescent cell death by inhibiting the PI3K-AKT pathway [251]. Furthermore, the antioxidant properties of the MedDiet can act on ROS by attenuating DNA damage and decreasing the excess number of senescent cells [42,68].

Regarding cancer, cellular senescence can protect tissues against tumorigenesis [270]. Indeed, anti-cancer therapies (chemotherapy or radiotherapy) induce senescence in cancer cells [271]. However, the persistence of therapy-induced senescent cells can be detrimental. Overall, a strategy that eliminates these persistent cells in the long term to minimize tumor progression and avoid adverse effects is of interest. Specifically, quercetin, in combination with dasatinib in aged mice, eliminated senescent cells and optimized cardiovascular function and survival [269,272]. However, in the elderly, senescent cells represent a high percentage of the cellular reserve, and this could jeopardize tissue structural integrity or affect vascular endothelial cells, leading to liver damage and the fibrosis of perivascular tissue with important repercussions on health [273,274].

In summary, the MedDiet may be useful as a senolytic tool, although more molecular studies are needed to clarify the synergistic action of the various anti-aging foods on tissue senescent cells.

#### 4.1.8. Stem Cell Exhaustion

Stem cells in humans have the capacity for self-renewal and differentiation in various tissues [275]. A decrease in the regenerative capacity of tissues is characteristic of aging [5]. This is a consequence of intrinsic and extrinsic causes that generate a vulnerable scenario for stem cell preservation in all human tissues [276]. This scenario includes reduced cell-cycle activity in longer-lived stem cells [277], accumulated DNA damage [277], the overexpression of p16 INK4a (cell cycle inhibitory) proteins [278], and telomere shortening [279,280]. In addition, an optimal balance between the activation of cell regeneration and the inactivation of the cellular process is essential for proper cell function. Therefore, circuits that safeguard progeroid stem cells’ quiescence, such as INK4 induction and IGF-1 depletion, are essential [5]. Overall, the deterioration in stem cell regenerative capacity and its lack of control contribute to aging and increase the risk of age-related diseases. In the case of hematopoietic tissue, the regenerative potential diminishes with age, and immunosenescence occurs. This phenomenon can often favor subclinical inflammation, which contributes to the development of age-related diseases [281].

Some components of the MedDiet, in combination or separately, have shown benefits in attenuating stem cell depletion. In the work of Cesari et al., adherence to the MedDiet in very old people was associated with increased numbers of endothelial progenitor cells [282]. Endothelial stem cells are essential for maintaining vascular homeostasis and renewing injured vascular cells [283,284]. Thus, the MedDiet intervenes in the early stages of the atherosclerotic process, which could have important implications for the early prevention of CVD [30]. Particularly, oleuropein and oleacein play a protective role in the senescence of endothelial progenitor cells induced by angiotensin II (a key pathological factor in hypertension) [285]. In addition, oleopurein stimulates osteoblastogenesis and the mineralization of the cellular matrix and inhibits bone resorption. An increase in serum osteocalcin levels was detected in elderly patients on the MedDiet enriched with VOO [286,287]. Overall, the risk of osteoporosis was reduced, including a bone protective effect in a two-year intervention study derived from PREDIMED [286].

In relation to carcinogenesis, an increased incidence of hematological malignancies has been associated with immunosenescence [288]. In this sense, multiple polyphenols in OO preserve hematopoietic stem cells and their differentiation [141,289]. Further, there is an increased risk of aggressive and invasive skin cancers (melanoma and basal cell carcinoma) associated with aging and exposure to ultraviolet radiation [290,291]. Both circumstances induce the premature senescence of fibroblasts and the activation of fibroblast-to-myofibroblast transitions [290,292]. These findings could favor fibrosis along with the loss of skin elasticity and an increased risk of oncogenesis. As an anti-aging and preventive therapy against hyperplasia and skin cancers, resveratrol can induce anti-inflammatory and antioxidant changes [293]. However, further studies are needed to conclude with certainty the benefits of resveratrol.

AD is included in the group of SASP or pathologies secondary to an altered secretome (growth factors, ROS, cytokines, and metalloproteinases). In fact, late astrocytes generate increased secretion of SASP factors that deregulate physiological functions. Dysfunctional astrocytes produce a chronic inflammatory response and pathologies of the central nervous system. In this context, altered synaptic plasticity, blood–brain barrier impairment, and glutamate excitoxicity with decreased neural stem cell proliferation have been observed [257,294]. As a preventive option to avoid this pathological condition, studies with DHA plus EPA supplementation have shown beneficial effects in patients with mild AD [295,296]. Therefore, MedDiet may be useful, as omega-3 PUFA-rich components are frequently ingested. Similarly, this consumption has shown a protective effect against the incidence of AD [296]. Flavonoids from cereals, vegetables, fruits, and OO had potentially beneficial effects, including free-radical scavenging, anti-inflammatory effects, and protection against Aβ neurotoxicity [297,298,299]. Further, the positive influence of polyphenols on cerebrovascular health is considered relevant, including lipoprotein oxidation, platelet aggregation, and endothelial cell reactivity [300].

In conclusion, MedDiet can be useful in the prevention of prevalent age-related diseases thanks to foods such as OO, which can palliate or slow down the deterioration of stem cells. However, more studies are needed to contrast this approach, as there are controversies on some points.

#### 4.1.9. Altered Intercellular Communication

Cellular coordination is essential for proper functionality. Different soluble molecules allow intercellular communication: cytokines, chemokines, growth factors, and neurotransmitters [301]. Multiple intercellular, endocrine, and neuronal pathways undergo alterations in the aging process. Specifically, neurohormonal signaling is altered in aging as inflammatory reactions increase and immune system reactivity to pathogens and precancerous cells decreases [5]. Aging is related to inflammation, one of the most relevant intercellular communication processes, and this association is multicausal. Among the causes are the accumulation of proinflammatory tissue damage by the increased secretion of cytokines and adipokines, excess ROS, immunosenescence, increased activation of the NK-κB pathway, changes in the gut microbiome and intestinal permeability, and altered autophagy response [302,303,304,305,306]. Thus, the elderly presents a low-grade systemic inflammatory phenomenon that favors age-associated chronic diseases and increases the risk of mortality [307].

The MedDiet demonstrated anti-inflammatory effects in relation to different markers, such as interleukin 6 (IL-6) or tumor necrosis factor-alpha (TNFα) [38,308,309]. In this context, multiple in vitro studies have positioned OO polyphenols as sources of health-promoting properties [141]. Zhang et al. highlighted the anti-inflammatory properties of HT mediated by the suppression of cyclooxygenase-2 (COX-2) and the expression of inducible nitric oxide synthase [310]. HT also reduces superoxide ions and inhibits the excess of important inflammatory mediators in humans, prostaglandin E2, probably due to the reduced expression of COX-2 [311]. Thus, it is intuited that the ability of polyphenols to regulate the production of proinflammatory molecules may have a salutary impact on older people. In addition to HT, other polyphenols such as oleocanthal and oleouropein inhibit TNFα-induced matrix metalloproteinase 9 through the anti-inflammatory pathway shared with ibuprofen (a non-steroidal anti-inflammatory drug that inhibits COX-2) [253,312]. Further, the flavonoid apigenin can act as an immunomodulator and principal regulator of TNFα in lipopolysaccharide-induced inflammation [313].

Atherosclerosis is an inflammatory disease and is closely linked to endothelial dysfunction. Dell’Agli et al. showed that OO polyphenols slow down the expression of proatherogenic molecules through the inactivation of NF-kB in endothelial cells [314]. These polyphenols were also potent against ROS, prevented the oxidative damage of genetic materials, and enhanced the antioxidant power of endothelial cells [315]. In this sense, the study of Meza-Miranda et al. determined that a breakfast based on VOO favorably regulated the pathophysiological mechanisms of premature atherosclerosis in endothelial cells [316]. Camargo et al. demonstrated that a breakfast rich in OO could curb the expression of proinflammatory genes and favor a less harmful inflammatory profile [317]. In addition, EVOO enhances the anti-inflammatory effect of high-density lipoprotein cholesterol and increases age-related antiatherogenic activity [318].

Moreover, the inflammation of neuronal cells is a fundamental mechanism in neurodegeneration. Proinflammatory immune-mediated mechanisms are essential in the pathogenesis and progression of AD and PD [319]. MedDiet has been associated with a lower risk of developing AD and PD [320]. In addition, the positive effects of omega-3 PUFAs on AD are due to their antioxidant and anti-inflammatory power. However, these PUFAs tend to oxidize. Therefore, the antioxidant capacity of polyphenols may be of interest as adjuvants [29]. This is more supportive of adherence to the Mediterranean diet than the individual intake of a certain food that is commonly present in the MedDiet. Further, fruits and vegetables (including in the MedDiet), with great antioxidant and anti-inflammatory properties, reduce the risk of PD [321,322].

Regarding carcinogenesis, senescent tumor cells produce SAPS that control the senescence of neighboring cells [323]. Initially, secretome factors could prevent tumor progression or eradicate malignant cells except in situations where there is acute aggression to SASP [323]. At this point, some polyphenols, such as quercetin and phytosine, exhibit senolytic properties through the inhibition of the PI3K-AKT pathway [324,325]. Regarding the most prevalent cancers today, MedDiet, when applied to CRC, has shown a protective effect thanks to different compounds [326]. Among them, oleuropein is promising as a protective agent against colitis-associated CRC, and in mice with induced CRC and inflammatory cytokines such as TNF-α, IL-6, and COX-2 decreased [327,328]. Further, oleocanthal could reduce the risk of inflammatory bowel diseases and CRC [329,330]. Therefore, the inflammatory response is regulated by OO polyphenols as they inhibit NF-κB, and this implies the lower expression of different interleukins and COX-2. This microenvironment hinders tumor proliferation [331,332].

Therefore, the evidence supports the suggestion that the MedDiet has anti-inflammatory properties and, consequently, may positively influence the aging phenotype.

## 5. Aging, Mediterranean Diet and Microbiome

### 5.1. Microbiome

The gastrointestinal tract is colonized by an array of microorganisms, including bacteria, viruses, fungi, and protozoa. These coexist symbiotically with enterocytes without being identified by the immune system as pathogens [333]. These microorganisms make up the microbiota, which consists of a total of 52 different phyla and up to 35,000 species of bacteria, mainly Actinobacteria, Bacteroidetes, Firmicutes, and Proteobacteria [334].

The intestinal microbiota has its origin in the placenta, with low levels of non-pathogenic bacteria, mostly Bacteroidetes, and Firmicutes. After birth, the intestine of the newborn and infant is rapidly colonized. Factors such as the type of delivery (vaginal or caesarean) or the type of feeding (breastfeeding or formula feeding) are determinants of the microbiota [333]. During the first three years of life, the microbiota has low diversity. After the third year, the microbiota is similar to that of the adult stage. With aging, changes occur in the morphology and function of the microbiota. Thus, after 65 years of age, the microbiota experiences a decrease in Firmicutes and Bifidobacterium, with an increase in diversity for Clostridium [28].

The changes produced throughout life could alter the diversity of the microbiota, giving rise to metabolic and inflammatory alterations and causing the appearance of conditions such as inflammatory bowel disease or irritable bowel syndrome, among others [335]. Moreover, the microbiota has not only been linked to diseases of the gastrointestinal tract but also to other diseases such as obesity, diabetes, CVD, or cancer [336]. In fact, since the concept of the “gut–brain axis” was created, gut microbiota has also been linked to neurodegenerative diseases [337].

### 5.2. Interplay between Aging and the Microbiome

The mechanisms by which the microbiota change with age are not fully understood. It is known that, in aging, physiological changes occur, such as alterations in dentition or decreased digestion and absorption, and modifications of lifestyle conditions, such as hospitalization or nursing homes. These modifications could be responsible, in part, for changes in diet and thus for the nutritional status of the elderly [338]. Moreover, in aging, especially in respect of frailty, there is usually a reduction in the amount and variety of food, which leads to the appearance of malnutrition [339]. Diet seems to be one of the pillars of changes in the microbiota. The microbiota may modulate changes in aging-related to innate immunity, cognitive function, and sarcopenia, which are components of frailty syndrome [340]. In fact, recent studies have suggested that loss of the gut microbiota is more related to age-associated frailty than to chronological age [341].

During the transition from adult to elderly, the main changes in the intestinal microbiota occur. Microbial diversity decreases compared to young adults [342]. In elderly centenarians, the microbiota consists mostly of Bacteroidetes and Firmicutes. However, in comparison with young adults, there are changes in subgroups such as Firmicutes, with an increase in Bacilli and a decrease in Clostridium. In addition, there is also an increase in Protebacteria [343]. Intestinal dysbiosis mainly involves changes in the abundance of commensal bacteria, also including some that function as opportunistic pathogens. The importance of the dysbiosis phenomenon is that it stimulates the excretion of endotoxins, i.e., amyloid and microbial lipopolysaccharides, to promote intestinal wall permeability and increase the peripheral circulation of proinflammatory cytokines [344].

In the ELDERMET study, the microbiota was studied in elderly people living in a community or living in long-stay homes in Ireland [338]. In the first group, microbiota configurations were more affected by antibiotic use than the microbiota of individuals in long-stay residences. However, this first group presented greater recovery after antibiotic use. The second group showed a loss of microbial components associated with ill health and a gain in altered microbiota associated with aging [338]. These findings on the relationship between microbiota, diet, and health status are supported by Claesson et al. [342]. They demonstrated, through an analysis of the composition of the fecal microbiota separated in 178 elderly subjects, that a change in diet associated with a transfer to a nursing home caused a change in the composition of intestinal bacteria, which correlated with nutritional status, inflammatory markers, comorbidity, and frailty [342]. Thus, the aging process and other environmental factors may alter the composition of the microbiota and contribute to the development of chronic low-grade inflammation [7].

Therefore, maintaining diversity in the microbiota appears key to maintaining health status and preventing frailty.

### 5.3. Mediterranean Diet and Microbiome: Health Status and Disease

**Figure 3:**
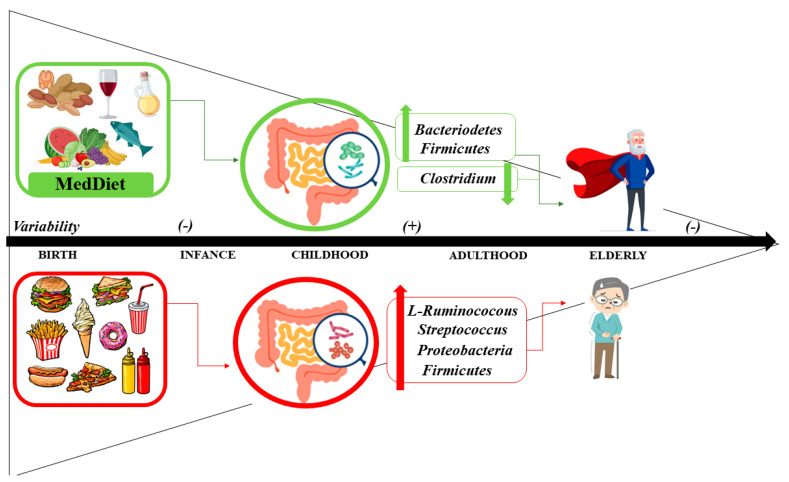
Influence of diet in gut microbiome related to aging.

Diet has a major impact on the biology of the gut microbiota [345]. Some nutrients have effects on the structure, function, and secretion of metabolites of the gut microbiota that can modulate immune functions and multiple metabolic and inflammatory pathways [346,347]. Emerging evidence is showing that adherence to the MedDiet promotes beneficial effects on the microbiota, favoring microbial diversity mainly in the colon, and it is associated with a reduction in Clostridium and an increase in Bacteroidetes and Firmicutes [28] (Figure 3).

#### 5.3.1. Mediterranean Diet, Microbiome and CVD, Obesity and Diabetes

Variations in the microbiota have been linked to the development of diseases. The microbiota can be modified through diet. In fact, polyphenols from the MedDiet play a key role in the microbiota. These compounds can reach the gut microbiota and modify the bacterial population and its metabolism. In this respect, it has been reported that the administration of polyphenols in rats, specifically resveratrol and curcumin, was associated with alterations in the Bacteroidetes and Clostridium groups of bacteria, thus providing metabolic benefits in glycemic control [348]. Additionally, the high content and bioavailability of fiber in the MedDiet (two times higher than in a Western diet) have beneficial effects on the cardiovascular system of older adults. These benefits could be due, in part, to changes in the microbiota. Fiber appears to have a positive impact on the composition of the gut microbiota, increasing the number of beneficial bacteria, inhibiting the growth of pathogens, and reducing atherogenic serum cholesterol in the microbiome. It also prevents glucose intolerance by reducing postprandial hyperglycemia through the formation of a viscous layer around the small intestine, which slows down the chyme transition [7]. This, in turn, increases the thickness of the aqueous layer through which solutes must pass to reach the enterocyte membrane, leading to a decrease in glucose in the enterocyte blood and resulting in a decreased absorption of glucose, lipids, and amino acids [7]. High fiber intake has been found to promote modifications of the gut microbiota with an increase in Bacteroidetes (in particular, *Bacteroides acidifaciens*), which produce high levels of short-chain fatty acids, including acetate, butyrate, and propionate [349]. Some of the beneficial effects of these metabolites are thought to be mediated by binding to specific G-protein-coupled receptors expressed on enteroendocrine and immune cells [349]. Conversely, poor adherence to the MedDiet was associated with an increase in l-Ruminococcus and Streptococcus bacteria and an increased concentration of trimethylamine N-oxide (TMAO) in urine. Compared to the Western diet, the MedDiet has significantly lower contents of choline and L-carnitine (present in egg, cheese, and red meat), and the production of TMAO by the microbiota has been shown to be lower [350]. This could reduce the risk of CVD, independent of the presence of cardiovascular factors. Zhu et al. concluded that an elevated level of TMAO could also be involved in the pathogenesis of obesity and type 2 diabetes mellitus, as it induces vascular inflammation and a prothrombotic effect by increasing platelet hypersensitivity to multiple agonists [351]. Indeed, the review by Cornejo-Pareja et al. concludes that the increase in fat mass in obese patients is not only due to more efficient energy uptake but that the microbiota is involved in changes in endotoxemia, intestinal permeability, insulin resistance, the hormonal environment, the expression of lipogenesis regulatory genes, interaction with bile acids and changes in the proportion of brown adipose tissue [352].

#### 5.3.2. Mediterranean Diet, Microbiome and Cancer

Numerous epidemiological studies have supported the importance of lifestyle factors and exposure to known or suspected carcinogens in the development of cancer. In fact, it is estimated that 30–35% of cancer risk factors are associated with diet, physical activity, and/or energy imbalance [353], and 15–20% of cancers are caused by infectious agents [354]. The microbiota that inhabits our body can be considered an environmental factor to which we are continuously exposed throughout life. However, the underlying mechanisms by which the MedDiet decreases the risk of cancer are not entirely clear [355]. In the diet–microbiota interaction, it has been observed that many dietary and digestive components are metabolized by bacteria in the gastrointestinal tract, leading to tumor suppressor metabolites and putative oncometabolites [356,357]. As an example, the excessive consumption of red meat, present in the Western diet, is a risk factor for CRC and other cancers by several mechanisms, including some that are dependent on intestinal bacteria. Elevated levels of protein intake can lead to an increase in certain types of bacteria, including Bacteroides and Firmicutes. These ferment amino acids into N-nitroso compounds, which induce DNA alkylation and mutations in the host [357]. Proteobacterias that encode nitroreductases and nitrate reductases are also related to this process, which is strongly associated with inflammation [358].

In addition, in the process of the digestion of saturated fat associated with red meat consumption, approximately 5% of the primary bile acids escape from the enterohepatic circulation and reach the colon, where they are converted by bacteria into secondary bile acids. Primary cholic acid is converted to secondary deoxycholic acid by certain bacteria, including *Clostridium scindens*. Secondary deoxycholic acid functions as a tumor promoter by disrupting cell membranes to release arachidonic acid, which is converted by cyclooxygenase-2 and lipooxygenase into prostaglandins and ROS that trigger inflammation and DNA damage [359]. By contrast, the dietary fiber present in the MedDiet is fermented by certain types of colonic bacteria, such as Clostridium groups IV and XIVa, into short-chain fatty acids. Butyrate, which is one of the most abundant short-chain fatty acids, is the main source of energy for colonocytes and is involved in the prevention of CRC. It has been observed that butyrate probably exerts its tumor-suppressive properties through multiple mechanisms. Butyrate epigenetically regulates the expression of genes that are involved in apoptosis and cell proliferation apoptosis [116]. It also acts as a ligand for certain G-protein-coupled receptors due to its involvement in tumor suppression [360]. Both mechanisms are believed to be important for butyrate’s ability to induce regulatory T cells. In addition, butyrate helps maintain the epithelial barrier function, which is important for preventing inflammation. Other components of the MedDiet that are related to cancer prevention are polyphenols. Ellagitannins are polyphenols found in nuts and berries. When they reach the intestine, they are modified by the microbiota and transformed into different compounds. Urolithin is one of the most studied products, and it has been shown that it can be absorbed by the enterohepatic circulation and transported by the blood and thus distributed to different tissues. It has anticarcinogenic effects through the inhibition of the Wnt signaling pathway, which could have a protective effect against CRC [361].

Thus, our diet dictates whether the microbiota produces metabolites that exacerbate or enhance tumor progression [362].

#### 5.3.3. Mediterranean Diet, Microbiome and Neurological Diseases

Regarding neurodegenerative diseases, better cognitive functions and a lower risk of dementia have been associated with higher adherence to the MedDiet. The PREDIMED study demonstrated a modest beneficial effect of adherence to the MedDiet for 4–6 years on cognitive functions in cognitively healthy adults at high risk of CVD, especially in the domains of global cognition, memory, and executive function [363,364]. By contrast, no benefit on cognitive function was reported after 1 year of the MedDiet in older adults in the NU-AGE trial. However, participants with higher adherence to the MedDiet demonstrated better global cognition and episodic memory compared with those that have low adherence [365]. These benefits appear to be related to certain components of MedDiet (omega-3 fatty acids, antioxidants, and polyphenols) as they may inhibit neuroinflammation associated with AD and other degenerative diseases [366].

Changes in the microbiota could also be involved in the pathogenesis of these diseases by initiating and perpetuating neuroinflammatory processes. In this respect, a study demonstrated the existence of the brain microbiota in cerebral blood vessels through micrographs of the human brain [367]. These bacteria and gut-derived toxins appear to compromise the integrity of the blood–brain barrier and could contribute to early neuroinflammatory changes by stimulating microglia and hindering amyloid clearance [368,369]. In addition, microbial amyloid and circulating liposaccharides activate innate resistance receptors, such as the Toll-like receptor and the receptor for advanced glycation end products, to increase proinflammatory signaling and to promote chronic neuroinflammation and progressive neurodegeneration, especially in sensitive brain regions such as the hippocampus [368,370]. Moreover, the microbiota has also been linked to other disorders, such as epilepsy. In fact, one study in epileptic patients found that antibiotic treatment reduced seizure frequency by 10% [371].

Nearly 60% of the variation in gut microbiota is attributable to diet [372]; therefore, modulation of the gut microbiota through diet could be an effective approach for reducing the inflammation associated with neurological diseases. Preliminary data have shown positive associations between the MedDiet and increased numbers of beneficial species of the microbiota, e.g., Bacteroidetes, and their short-chain fatty acid metabolites, which have anti-inflammatory effects [373,374]. However, only a few studies have evaluated dietary patterns and gut microbiota, most of them being observational, which prevents establishing causality [375,376]. Further research is therefore needed to understand the complex relationships between the gut microbiota and cognitive health and whether diet-induced effects are mediated by alterations in gut microbiota.

All of this is important because increasing evidence suggests that the reprogramming of gut microbial functions through long-term adherence to healthier diets can influence physiological responses to nutrients and other features of host biology that are critical to promoting health and longevity [377]. Thus, the modification of the microbiota through MedDiet could benefit the evolution and prognosis of these diseases.

## 6. Methods

Interventional studies involving animals or humans, and other studies that require ethical approval, must list the authority that provided approval and the corresponding ethical approval code. A comprehensive search of the literature published in PubMed from November 2022 was conducted to identify articles relating to MedDiet, microbiota, aging, and frailty. Search strategies were based on the following search terms: MedDiet, polyphenols, omega-3 PUFAs, healthy aging, hallmarks of aging, telomere length, microbiota, oxidative stress, mitochondrial function, inflammation, cellular senescence, anti-senescence compounds, frailty, and sarcopenia. A selection of articles published in English providing original human research, observational prospective and retrospective studies, randomized controlled trials, reviews, and meta-analyses were included.

In addition, we considered case series, single-case reports, editorials, research or original articles, letters to the editor, comments (on an article or from the editor), responses (to a comment, letter, or article), corrections, short reports, short communications, perspectives, opinions, and discussions. Priority was given to the largest studies and to the strongest available evidence and most recent studies.

## 7. Conclusions

There is an increasingly aging global population. However, the way to achieve healthy aging has not yet been fully elucidated. The loss of function and frailty syndrome associated with aging increases the vulnerability of the elderly and their propensity to disease. There are different molecular pathways or hallmarks involved in aging that bring us closer to understanding the deterioration associated with the senescence process, such as genomic instability, telomere attrition, epigenetic effects, proteostasis, nutrient-sensing pathways, mitochondrial dysfunction, cellular senescence, stem cell depletion, and altered intercellular communication. Likewise, microbiota disturbances seem to play a relevant role in frailty in the elderly.

It has been shown that MedDiet promotes healthy aging, increasing the life expectancy of the population. This review has shown that MedDiet positively influences the molecular pathways that determine age. Consequently, MedDiet has been associated with a lower risk of age-related diseases, mainly CVD, neurodegenerative, and oncological diseases. Therefore, further evidence of the beneficial effects of this dietary pattern on human health and longevity has been provided. However, most studies do not evaluate the impact of the Mediterranean diet pattern as a whole on the hallmarks of aging but rather its individual components, especially certain bioactive components. Certainly, there are some clinical trials exploring the role of the Mediterranean diet (mostly PREDIMED substudies), but they focus on specific dietary supplementation with nuts or EVOO. Therefore, it would be useful to evaluate the pattern as a whole without special emphasis on these more studied components. In addition, more quality studies on the MedDiet and the prevention of frailty and disease in aging are needed, as many studies are observational, and causality cannot be determined.

Overall, more research is needed to provide a better understanding of the mechanism of action of MedDiet on aging. However, at present, MedDiet could be recommended as a baseline anti-aging therapy to prevent frailty and maintain functionality until the later stages of life, as the benefits of MedDiet on human health present robust evidence.

## Data Availability

Not applicable.
